# ALVIS: interactive non-aggregative visualization and explorative analysis of multiple sequence alignments

**DOI:** 10.1093/nar/gkw022

**Published:** 2016-01-26

**Authors:** Roland F. Schwarz, Asif U. Tamuri, Marek Kultys, James King, James Godwin, Ana M. Florescu, Jörg Schultz, Nick Goldman

**Affiliations:** 1 European Molecular Biology Laboratory—European Bioinformatics Institute, Wellcome Genome Campus, Hinxton, CB10 1SD, UK; 2 Science Practice, 83–85 Paul Street, London, EC2A 4NQ, UK; 3 Center for Computational and Theoretical Biology and Department of Bioinformatics, University of Würzburg, Biocenter, Am Hubland, 97074 Würzburg, Germany

## Abstract

*Sequence Logos* and its variants are the most commonly used method for visualization of multiple sequence alignments (MSAs) and sequence motifs. They provide consensus-based summaries of the sequences in the alignment. Consequently, individual sequences cannot be identified in the visualization and covariant sites are not easily discernible. We recently proposed *Sequence Bundles*, a motif visualization technique that maintains a one-to-one relationship between sequences and their graphical representation and visualizes covariant sites. We here present Alvis, an open-source platform for the joint explorative analysis of MSAs and phylogenetic trees, employing *Sequence Bundles* as its main visualization method. *Alvis* combines the power of the visualization method with an interactive toolkit allowing detection of covariant sites, annotation of trees with synapomorphies and homoplasies, and motif detection. It also offers numerical analysis functionality, such as dimension reduction and classification. *Alvis* is user-friendly, highly customizable and can export results in publication-quality figures. It is available as a full-featured standalone version (http://www.bitbucket.org/rfs/alvis) and its *Sequence Bundles* visualization module is further available as a web application (http://science-practice.com/projects/sequence-bundles).

## INTRODUCTION

Visualization and explorative analysis of multiple sequence alignments (MSAs) are essential to all areas of computational biology. Different methods for the graphical identification of sequence motifs have been proposed over the years. The most popular are *Sequence Logos* (1), *HMMLogos* (2) and, recently, *pLogos* (3). These are aggregative or consensus-based visualizations of MSAs: alignment columns are summarized by individual characters scaled according to their relative frequencies or statistical significance.

**Figure 1.**
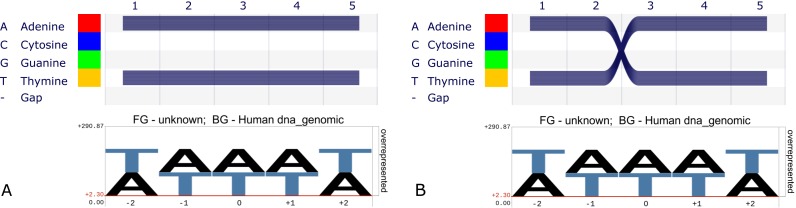
(**A**) *Sequence Bundles* (top) and *pLogo* (bottom) representation of an alignment of 1000 sequences with 500 instances of *AAAAA* and 500 instances of *TTTTT*. (**B**) The same visualizations rendered on a 1000 sequence alignment with 500 instances of *AATTT* and 500 instances of *TTAAA*. The *pLogo* representations in (A and B) are identical and reflect only the identical sitewise nucleotide frequencies in the two examples. (The different ordering of letters is a result of the chosen genomic background [human whole-genome].) *Sequence Bundles* clearly show the two sequence motifs in each case, because the nucleotides remain connected in the visualization.

While providing a compact view of large MSAs, the aggregative nature of logos is also a major limitation: individual sequences cannot be identified in the final graphical representation. Consequently, information about covariance between sites (residues or nucleotides) is lost. For example, consider the following two illustrative alignments. The first alignment consists of 500 copies of each of the sequences *AAAAA* and *TTTTT* (Figure 1A, bottom). The second alignment consists of 500 copies of each of the sequences *AATTT* and *TTAAA* (Figure 1B, bottom). Because the site-specific nucleotide frequencies are identical between the two alignments, aggregative motif visualization methods fail to capture the sequence motifs. Therefore, sequence logos are in general not able to help in the identification of specificity determining sites and correlated substitutions. The importance of residue–residue dependencies for the understanding of the evolution of protein function was pointed out as long ago as 1970 (4). The term ‘covarion’ was coined to denote concomitantly variable codons. The relevance of these correlated changes for the structure of proteins has been proven (5), and different methods relying on them for the prediction of protein structure were developed. For a recent review see [(6)] and references therein. In short, deliberately omitting information about correlation between sites in MSAs drastically reduces the information available about the evolution, structure and function of protein families.

To address this shortcoming, we recently developed *Sequence Bundles* (7), a novel visualization method based on stacked semi-opaque Bezier curves. *Sequence Bundles* form a grid with the sequence alphabet on the y-axis, ordered according to various biochemical properties, and the sequence positions on the x-axis. Grid cells are connected by semi-opaque curves (*threads*), one for each sequence in the alignment. In contrast to *pLogo*, the sequence motifs are now clearly visible (Figure 1A and B, top).

*Sequence Bundles* retain sequence identity, as every sequence has its own graphical representation (the thread). The one-to-one relationship between sequences and their visualization make them a powerful tool for explorative analyses of MSAs. We previously developed *CAMA*, an unsupervised ordination method for MSAs to detect statistical dependencies between sequences and sites in an MSA (8). *Sequence Bundles* show particular synergy with this method: sequences and sites may be repeatedly selected and their relationships are visualized in the bundle. This is of particular interest in phylogenetics, where such explorative analyses can be used to identify sequence motifs shared between monophyletic clades of a tree (synapomorphies), or where sequence motifs shared between paraphyletic clades (homoplasies) might indicate convergent evolution.

We now present *Alvis*, a platform for the joint explorative analysis of MSAs and phylogenetic trees which uses *Sequence Bundles* as its main visualization method. *Alvis* facilitates the identification of functional residues, detects correlated substitutions between distant sites, and helps find and visualize sequence motifs. Below we present an overview of the capabilities of the software and illustrate its use in a series of real-world case studies.

## MATERIALS AND METHODS

*Alvis* is implemented in Java and is available for all commonly used platforms. It imports and exports common sequence and tree formats as supported by the BioJava suite (9). Sequences can be loaded pre-aligned or can be aligned by the software using the EMBL-EBI Prank web service (10). Custom sequence alphabets are fully supported for analysis of sequential datasets beyond amino acid and nucleotide sequences (e.g. copy-number profiles). Sessions can be saved and restored using the *File*−>*Open* and *File*−>*Save* commands. All settings and options are saved between sessions in a custom configuration file *∼.alvis*.

*Sequence Bundles* are implemented using custom code and the hardware-accelerated Java2D API. Generated images can be exported in high-resolution PNG bitmaps as well as in SVG vector format for later editing.

Tree reconstruction is performed using the fast NINJA neighbour-joining implementation (11). Tree visualization is achieved using the animated tree rendering framework PhyloWidget (12).

Profile Hidden Markov Model implementation is based on the BioJava library (9). Fisher Scores and CA are computed using a custom algorithm as presented in the CAMA method article (8). Sequence classification and feature detection are enabled through the integrated R gateway using rJava/JRI (13) and *kernlab* (14).

*Alvis* is open source under GNU Affero GPL v3.0 and can be downloaded from https://bitbucket.org/rfs/alvis. *Sequence Bundles* visualization is also available online at http://science-practice.com/projects/sequence-bundles and http://www.ebi.ac.uk/goldman-srv/sequencebundles.

## RESULTS

**Figure 2.**
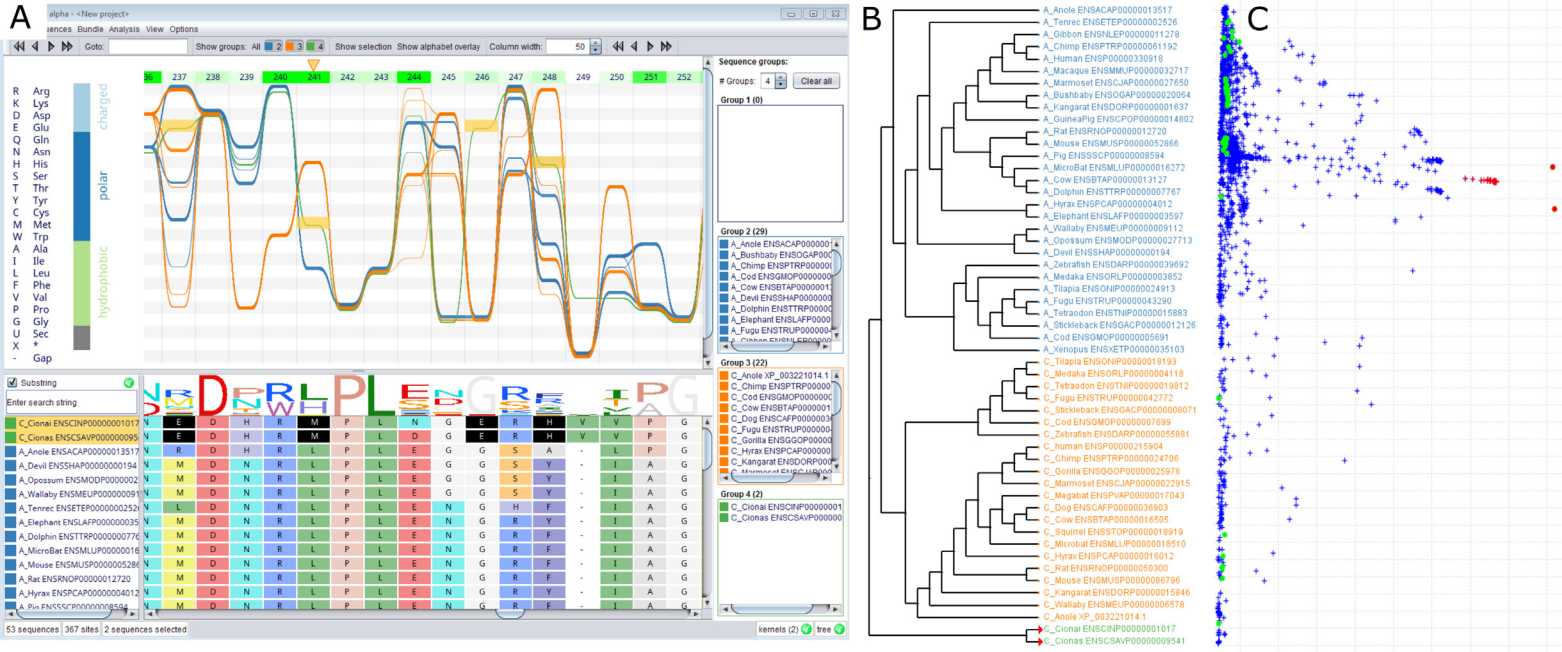
(**A**) *Alvis*'s *Sequence Bundles* visualization of the haloacid dehalogenase family. The bundle shows three sequence groups in different colours. Horizontal dependencies are immediately visible. For example, all *Ciona* sequences (selected in red) have a Met in position 241 and also exclusively have a Glu residue in position 246 and a His at position 248. This information is not available from the standard sequence logo (below). Above the bundle, green shaded markers indicate which sites are most likely responsible for the grouping. In agreement with the original paper (23), site 241 (marked with an orange triangle) is found as being most significant. (**B**) *Alvis*'s rendering of the associated phylogenetic tree. The group colours match those in the bundle. (**C**) CA scatterplot computed by *Alvis* on the same MSA. Sequences are displayed as green points, sites as blue crosses. Selection of sites and sequences in this plot (red) induces the highlighting of the corresponding sites and sequences in the alignment and bundle views. Residue Met-241 is identified as significantly associated with *Ciona*.

*Alvis* combines the traditional alignment and sequence logo view with a *Sequence Bundles* representation of the underlying MSA (Figure 2A). The alignment and bundle views are synchronized so changes to magnification, position in the alignment or selections made in one are reflected in the other. Sequences and sites can be selected manually or by entering queries in a search box using a simple query language. The query language accepts arbitrary boolean combinations of regular expressions on both the sequence labels as well as the actual sequences.

All aspects of the bundle rendering engine are customizable, including curvature, group colours and cell dimensions. Results can be exported to produce visually appealing publication-quality figures.

### Phylogenetic analysis

*Alvis* imports phylogenetic trees or builds its own tree based on pairwise distances computed from an evolutionary sequence kernel (15) derived from the MSA. Tree visualization (Figure 2B) includes standard layout schemes (circular, rooted, unrooted) and layout operations (rerooting, node flipping, etc.). The tree visualization engine is linked to the alignment and bundle views to reflect selections made by the user. In the tree view, sequences can be selected based on individual taxa (leaf nodes), clades and sub-clades.

Sequences can be assigned to groups, each of which may be given a unique colour which is applied to its members’ threads for easy identification of groups in the bundle. Assignment into groups may be performed manually, by dragging and dropping selected sequences into a group container, or automatically using spectral clustering (16).

### Numerical analysis

A typically fundamental task when analysing MSAs is the identification of the main sequence clusters and the sites they have in common. This can be achieved in a supervised or unsupervised manner, both of which require a numerical representation of the sequence data. *Alvis* can train a profile HMM (17) on the MSA and will use the Fisher scores of the emission probabilities (18) as a numerical embedding. In the supervised scenario (i.e. given a set of user-defined groups) the *detect sites* feature trains one support vector machine (SVM) classifier (19) per site. Leave-one-out cross validation is performed on each SVM to identify sites that best explain the chosen grouping. Complementarily, correspondence analysis (CA) is an unsupervised ordination method to detect sequence groups and the sites that define the grouping (8). In *Alvis* it is based on an interactive scatterplot of both sequences and sites (Figure 2C), which again is linked to the alignment, bundle and tree views. Selecting a cluster of points in the CA plot highlights the corresponding sequences and sites in the bundle as well as the alignment (see Figure 2), allowing for systematic and interactive exploration of the MSA. All kernel matrices and the Fisher scores can be exported for analyses elsewhere.

In combination, the alignment view, bundle visualization, phylogenetic reconstruction and numerical analyses form an ideal toolkit for explorative analyses of MSAs. We present three example applications, each chosen to illustrate how *Alvis* facilitates scientific discovery not readily possible using other existing methods.

For a tutorial on how to use Alvis, please refer to our introductory video at https://vimeo.com/146710536.

### Identification of specificity-determining sites in two HAD phosphatases

Mammalian haloacid dehalogenase (HAD)-type phosphatases are an ancient protein family. More than 40 enzymes with important functions in physiology and disease are encoded in the human genome (20,21). One member of this family, chronophin, regulates cofilin-mediated actin reorganization by dephosporylating phospho-serines (22). Surprisingly, its closest paralogue, the aspartate-based, ubiquitous, Mg^2 +^-dependent phosphatase (AUM), functions as a tyrosine-phosphatase (23).

Here, we demonstrate computational detection of specificity-determining sites exclusively using features implemented in *Alvis*. We imported an alignment of 53 sequences comprising the chronophin and AUM paralogues across vertebrates as well as *Ciona* as an additional outgroup (23)(Figure 2A). We reconstructed an evolutionary tree using an alignment kernel (15) based on the BLOSUM62 substitution matrix, which correctly recovered the paralogue structure and most phylogenetic relationships (Figure 2B). We assigned the two paralogue families and the outgroup to three sequence groups: AUM (blue), chronophin (orange) and *Ciona* (green). The visualization clearly shows dependent sites. For example the *Ciona* sequences have a Met in position 241 and also exclusively have a Glu residue in position 246 and a His at position 248. This information is lost in the traditional sequence logo view (Figure 2A, below the bundle).

To identify sites that distinguish best between the defined sequence groups, we used the ‘detect sites’ feature implemented in *Alvis*. Green markers, whose opacity increases with decreasing cross-validation error, display these results and indicate which sites are most likely responsible for the grouping. *Alvis* identified site 241, containing Leu in AUM and His in chronophin, as most significant (Figure 2, site 241, marked with an orange triangle). CA (Figure 2C) confirms these findings. It first correctly identifies *Ciona* as most divergent (first principal axis). The sites that are spatially co-located with the two *Ciona* species in the scatterplot (selected in red) include Met-241. Selection in the scatterplot automatically selects the respective sequences and sites in the alignment and bundle views. Seifried *et al*. experimentally verified that introduction of a His residue at position 241 in AUM transfers chronophin-like substrate recruitment onto AUM (23). This example shows how *Alvis* enables the detection of specificity-determining sites with just a few mouse clicks. This example was also the basis for our introductory video at https://vimeo.com/146710536.

### Correlated substitutions in nucleotidyl cyclases

Cyclic nucleotides like adenosine 3′-5′ cyclic monophosphate (cAMP) and guanosine 3′-5′ cyclic monophosphate (cGMP) are small-molecule secondary messengers that play a key role in intracellular signalling. They are synthesized by nucleotidyl transferases which can be assigned to two groups based on their substrate specificity. While guanalylate cyclases take guanosine triphosphate (GTP) as a substrate, adenylate cyclases use adenosine triphosphate. Substrate specificity is defined by two positions: mutating Lys to Glu (pos. 146) and Asp to Cys (pos. 305) in a guanylyl cyclase switches its catalytic activity to an adenylyl cyclase (24). This feature made the nucleotidyl cyclases a common study object for the automated identification of specificity-determining sites (e.g.(25–29)).

**Figure 3.**
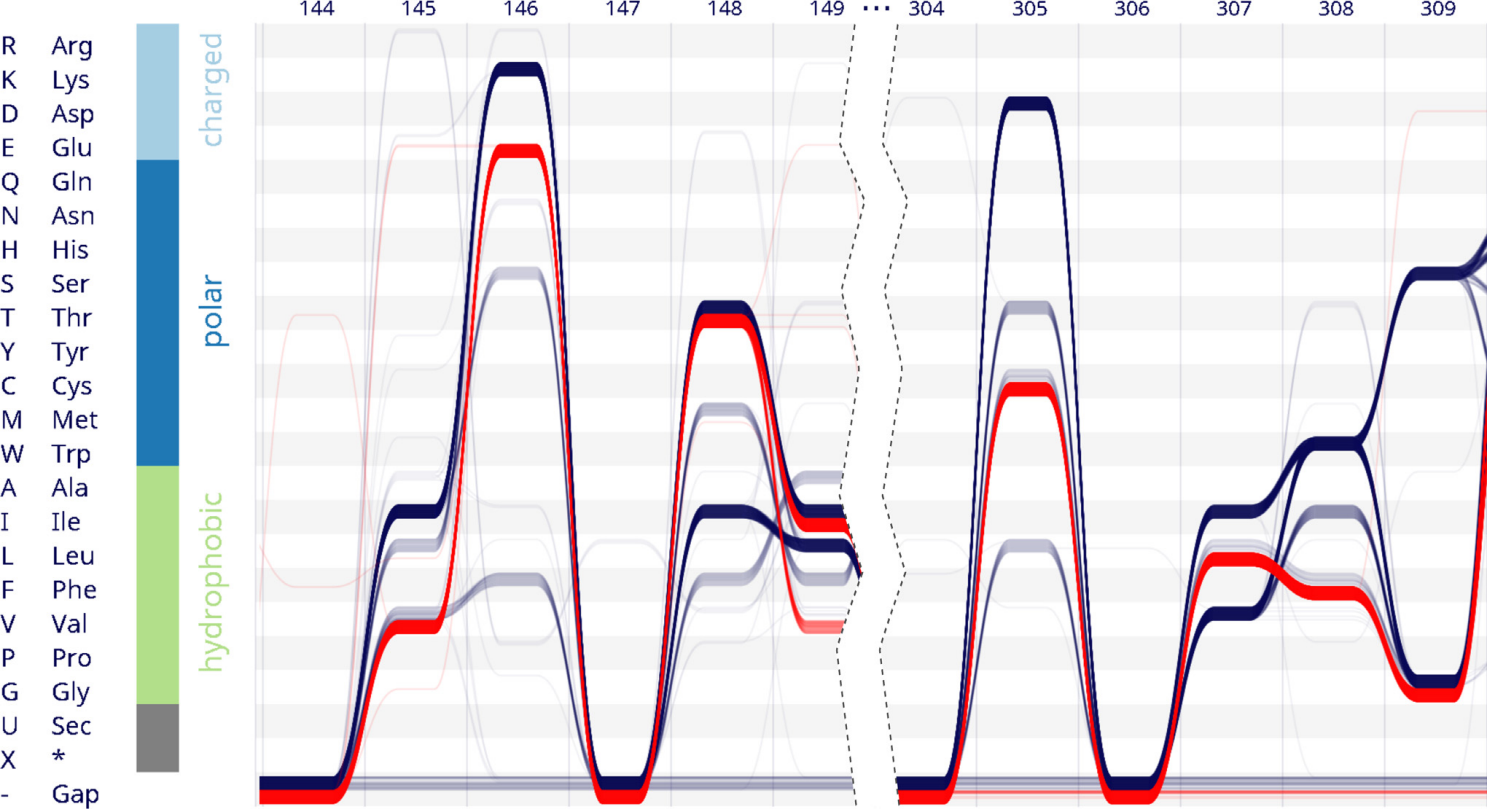
*Alvis* visualizes an alignment of 1224 mammalian nucleotidyl cyclases. Sequences containing Glu in position 146 are selected (red). None of these sequences contain Asp within the functionally correlated site 305. Further differently conserved sites like 307 and 308 also become apparent.

We use *Alvis* to test whether a correlation between the two specificity-determining sites is conserved throughout the Mammalia. We extracted all mammalian nucleotidyl cyclases annotated by the *Pfam* database (30) (ID:PF00211). The sequences were aligned to the corresponding HMM using *Pfam*'s web service. The resulting alignment comprising 1224 protein sequences was loaded into *Alvis* (Figure 3). In agreement with the experimental results, the visualization shows that the relevant sites (146 and 305) are dominated by two variants each. *Alvis* enables us to select all sequences containing Glu in position 146 with a single mouse click (Figure 3, threads coloured red). In contrast to a standard sequence logo, the bundle view reveals that none of these sequences contains Asp in position 305; instead, all have either Cys or a gap. Thus, a correlation characterised experimentally in a single protein is seen to be transferable to all sequenced mammalian nucleotidyl cyclases. This example illustrates how *Alvis* can condense information encoded in an alignment of more than 1000 sequences and still represent key characteristics of the underlying sequences.

### Comparison to *pLogo* in the CaMKII motif detection task

Recently O'Shea *et al*. proposed *pLogo*(3), a variant of a conventional sequence logo that scales the height of the characters using a probability model. Additionally, it can create an alignment logo conditional on individual residues at user-specified sites (so-called ‘fixing’ of sites). This partially overcomes the lack of visualization of horizontal dependencies in traditional sequence logos, because the user can iteratively select each site, restrict attention to each residue observed, and see how the remaining logo changes.

**Figure 4.**
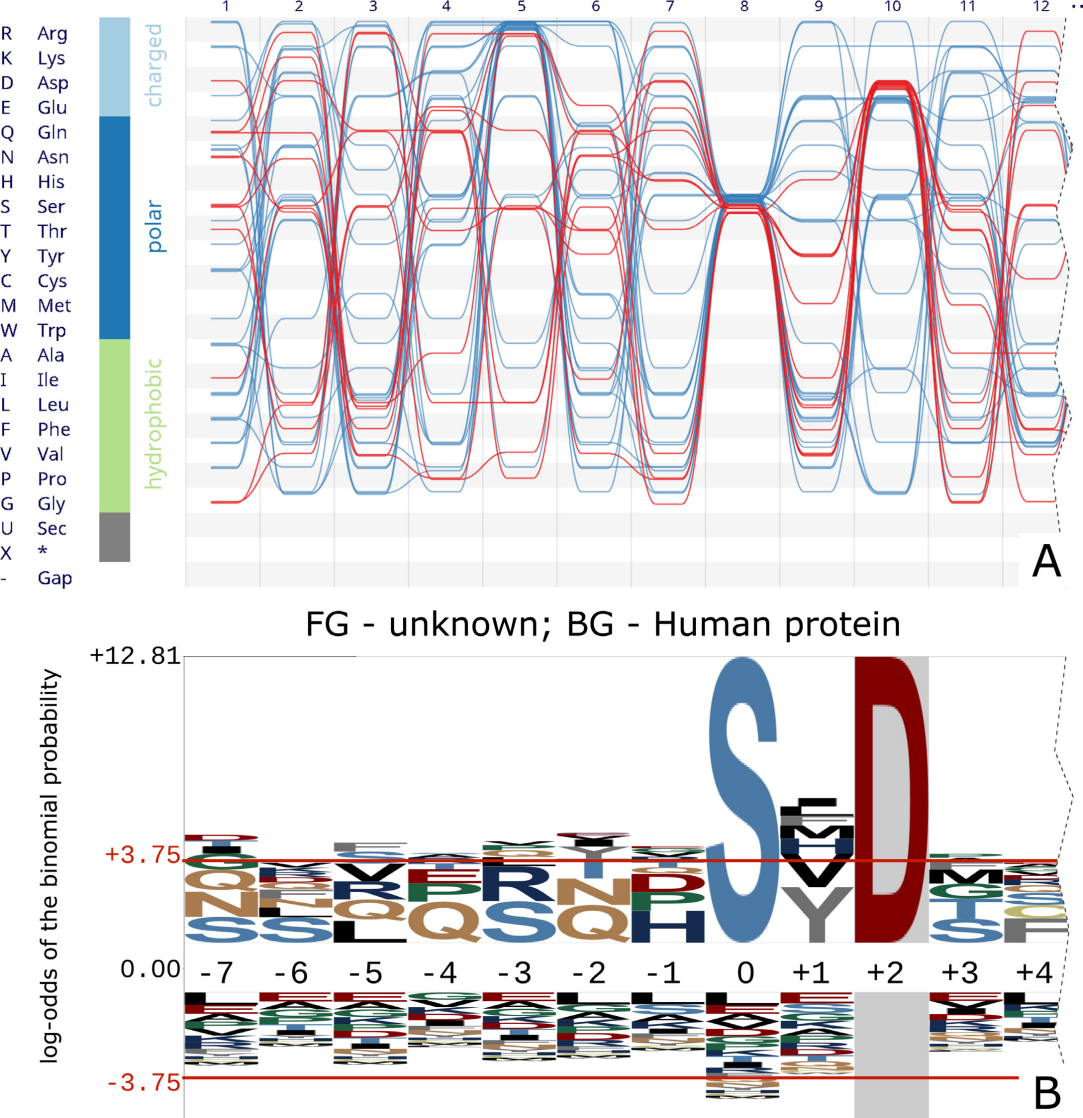
(**A**) The visualization of horizontal dependencies is a powerful tool to quickly investigate an alignment for co-dependent sites. Asp-10-containing sequences of calmodulin-dependent protein kinase II show no co-clustering at any of the other non-fully conserved positions. A strong preference for Asp-10 sequences to not have an Arg at position 5 however is visible and statistically significant (binomial test *P*-value 0.0053). (**B**) Representation of the same sequences with the *pLogo* software. In this version Asp-10 is ‘fixed’ (or conditioned on), also showing that there is no correlation between Asp-10 and position 5. However, the preference for avoiding Asp-5 remains hidden. The unfixed version (not shown) fails to capture the sequence motif altogether.

Figure 4 shows the calmodulin-dependent protein kinase II (CaMKII) sequences taken from (3). Three sites show a degree of conservation: site 5 dominantly shows an Arg residue, site 8 exclusively a Ser residue and site 10 shows amongst others an enrichment for Asp. O'Shea *et al*. used their ‘fixing’ of sites (3) to investigate whether the sequences with Asp at site 10 also have an Arg at site 5. They concluded there is no dependency or correlation between amino acid distributions at sites 5 and 10 (Figure 4B).

By retaining individual sequence information rather than site-wise averages or counts of residue frequencies, this last result is instantly visible from the visualization in *Alvis*. By clicking a grid cell in the bundle view all sequences containing the corresponding residue are selected in the alignment and marked in red in the bundle (Figure 4A). It is evident that sequences containing an Asp residue at site 10 show no conservation in other parts of the sequence, the red threads of the bundle being widely spread out at all other positions (i.e. not notably conserved). It is however evident from the *Alvis* representation that sequences with an Asp-10 residue seem to avoid Arg-5 (binomial test, *P*-value 0.0053). This information is lost in the ‘fixing’ method of *pLogo* when restricting the analysis to Asp-10 sequences only.

## DISCUSSION

While many improvements to traditional sequence logos have been proposed, *Alvis* provides a unique combination of interactive analysis capabilities and non-aggregative visualization. Figure 1 shows that *pLogo* is in essence a classical sequence logo with a different scaling on the *y*-axis. It suffers from the drawback typical of all aggregative methods: individual sequences cannot be identified in the final visualization. *Alvis* allows users to interactively explore MSAs on a consensus level as well as on the level of individual sequences. Because residues in the same sequence remain connected in the visualization, multi-site sequence motifs become visible even between distant sites. These multi-site motifs can also be investigated in the context of groups of sequences, by finding shared motifs or contrasting groups against one another. As often residues are exchanged based on functional constraints, such as hydrophobicity, the different y-axis legends available in *Alvis* induce sorting of residues according to biochemical properties. This helps to identify motifs conserved through functional similarity, not sequence.

Other visualization techniques have been proposed over the years which are capable of visualizing correlations between sites. Traditionally, DotPlots (31) have been used to this end, although they are best-suited to pairwise comparisons and are difficult to use for MSAs. Modern approaches include StickWRLD (32), a circular 3D visualization technique that uses coloured sticks to connect correlated residues. *Sequence Bundles*, while similar to a degree, uses a 2D approach approach of parallel lines, inspired by the more general parallel coordinate representations (33).

The ability to relate the MSA and any motifs visualized (whether using *Sequence Bundles* or other methods) to a realistic phylogeny is vital for a full understanding of the evolutionary history of the sequences and their associated functions (10). In addition, it helps in the assessment of the significance of motifs, which may be affected by factors such as low numbers of observations (few, highly divergent sequences) or data redundancy (domination by many closely related sequences). Others have proposed pseudo-counts and reweighting strategies to try to overcome these drawbacks (34). Pseudo-counts are already part of the HMM training in *Alvis* and we will consider applying both strategies to the visualization as well in a future version of *Alvis*.

Gaps in MSAs carry significant evolutionary information, particularly between divergent sequences (15,35). *Sequence Logos* and *pLogo* do not visualize or model gaps in the alignment. In *Alvis* we have implemented support for gaps both in the visualization and the numerical analyses, thus extending its potential applications beyond highly conserved alignments.

Often sequence motifs are investigated in an evolutionary context, for example when searching for evidence of convergent evolution. By mapping sequences to a phylogenetic tree one can detect evolutionary motifs that define monophyletic clades (synapomorphies) or contradict them (homoplasies). The latter can then be analysed for patterns of convergent evolution.

Sequence logos and bundles can both compactly visualize MSAs with a large number of sequences. *Alvis* additionally provides dimension reduction techniques for MSAs with a large number of sites using numerical ordination methods. This has proven to be useful when dealing with large proteins, multi-locus alignments or genomic profiles. CA has been shown to be an effective tool in identifying distant covariant sites and in providing a lower-dimensional representation of the data (8). Here, in contrast to some methods for the identification of correlated mutations (36), CA is an explorative analysis technique. It uses a chi-square metric instead of a euclidean metric in an algorithm related to principal-component-analysis to discover associations between sequences and sites.

Not all possible use cases can be foreseen and often researchers need to run bespoke algorithms on their alignments. Rather than implementing popular algorithms yet again, *Alvis* can connect to an existing R installation for advanced statistical analyses. For example, the *detect sites* function is by default based on supervised classification suitable for all kinds of sequence alphabets. Using the R interface, it could easily be augmented with specialized algorithms for detecting specificity determining sites in protein sequences, such as the evolutionary trace method (37). For a recent review, see also (38).

Alvis further provides export functionality for all internal data, the Fisher scores and kernel matrices. In the future, an extended R API will allow the user to push and pull data directly in and out of *Alvis* from their own R workspace.
